# Case of Primary Breast and Ipsilateral Axillary T-Cell Lymphoma: a Rare Occurrence

**DOI:** 10.1155/2020/6927835

**Published:** 2020-09-22

**Authors:** Faryal Afridi, Garry D. Ruben, Eric Oristian

**Affiliations:** ^1^George Washington University Hospital Program, 900 23rd Street NW, Washington DC 20037, USA; ^2^Chief Department of General Surgery, Holy Cross Hospital, 1500 Forrest Glen Rd. Silver Spring MD 20910, USA; ^3^Chief Department of Breast Surgery, Holy Cross Hospital, 1500 Forrest Glen Rd. Silver Spring MD 20910, USA

## Abstract

**Background:**

Malignant lymphomas of the breast are rare and can be primary or secondary. Non-Hodgkin Lymphoma involving the breast is even rarer comprising 0.04-0.5% of all breast malignancies (Takemura). The incidence is even lower for T-cell lymphomas compared with B-cell subtype. We report the rare incidence of primary T-cell lymphoma involving both breast and ipsilateral axilla.

**Case:**

This is the case of an 80-year-old female who initially presented with asymmetry of her right breast. Initial mammograms were inconclusive. MRI could not be performed due to the patient's severe claustrophobia. The patient was then lost to follow-up but re-presented with a new palpable density in the same breast. Subsequent mammogram showed a suspicious lesion with suspicious right axillary lymphadenopathy. Core biopsy was consistent with T-cell lymphoproliferative disorder involving both the breast and the axilla. She was then referred to medical oncology for management.

**Conclusion:**

Although rare, lymphoproliferative disorders of the breast can be encountered during workup for suspicious breast lesions. It is imperative that the surgeon is aware of this rare diagnosis to facilitate appropriate therapeutic intervention.

## 1. Introduction

Primary breast lymphoma (PBL) is defined as lymphoma initially presenting in breast tissue without a prior diagnosis of lymphoma. It is an extremely rare subtype of Non-Hodgkin lymphoma (<1% of all lymphomas) and represents only 0.04-0.5% of all malignant breast tumors [1] and 1.7-2.2% of all extra-nodal lymphomas [2]. The diagnostic criteria for PBL as laid down by Wiesman and Liao in 1972 is still the standard definition for this rare disease and comprises of adequate histopathological tissue for diagnosis, close relation between the lymphomatous infiltrate and breast tissue, no evidence of widespread or prior extramammary disease and ipsilateral lymph nodal involvement (if it arises simultaneously with the primary) [3]. It is postulated that PBL originates from two sources, either migratory lymphocytes present within mucosa-associated lymphoid tissue (MALT) or from lymphocytes residing inside the intramammary nodes [4].

An almost exclusive disease of the female breast, the median age of the presentation of primary breast lymphoma is 60-65 years [5]. The most common subtype of breast lymphoma is CD20 positive diffuse large B-cell lymphomas (50%) with other subtypes being follicular (15%), MALT 12.2%, Burkett/Burkett's like lymphomas (16.3%) [6]. The incidence of T-cell lymphomas is low (12-15% of all non-Hodgkin's Lymphomas) [7, 8], and this incidence is even lower in the breast even in large published series [3, 9, 10]. There is extensive reporting in the literature of T-cell lymphomas arising in association with breast implants especially anaplastic large cell lymphoma ALCL [11–16]. Roden et al. [17] in their paper published 2008 postulated a pathogenic hyperstimulation of T lymphocytes by the silicone component of breast implants which results in clonal expansion.

We present the case of primary breast and ipsilateral axillary T-cell lymphoma presenting in an 80-year-old female.

## 2. Case

This 80-year-old female with a history of hypertension, diverticulitis, and coronary artery disease was seen in our office after a suspicious lesion/area was seen on screening mammography and subsequent diagnostic mammogram. Due to extremely dense breast tissue, these images were reported to be inconclusive with slightly asymmetrical nodules seen within the right retro-areolar region labelled as BIRADS 3. MRI was recommended for further evaluation however due to severe claustrophobia patient could not have it performed. Her two subsequent mammograms were inconclusive as well, and the patient missed her one-year follow-up appointment for physical examination. She then re-presented to our office with a new palpable lesion in her lower outer right breast which was redemonstrated on physical examination in the office. She was referred for a diagnostic mammogram and ultrasound at this point.

**Figure 1:**
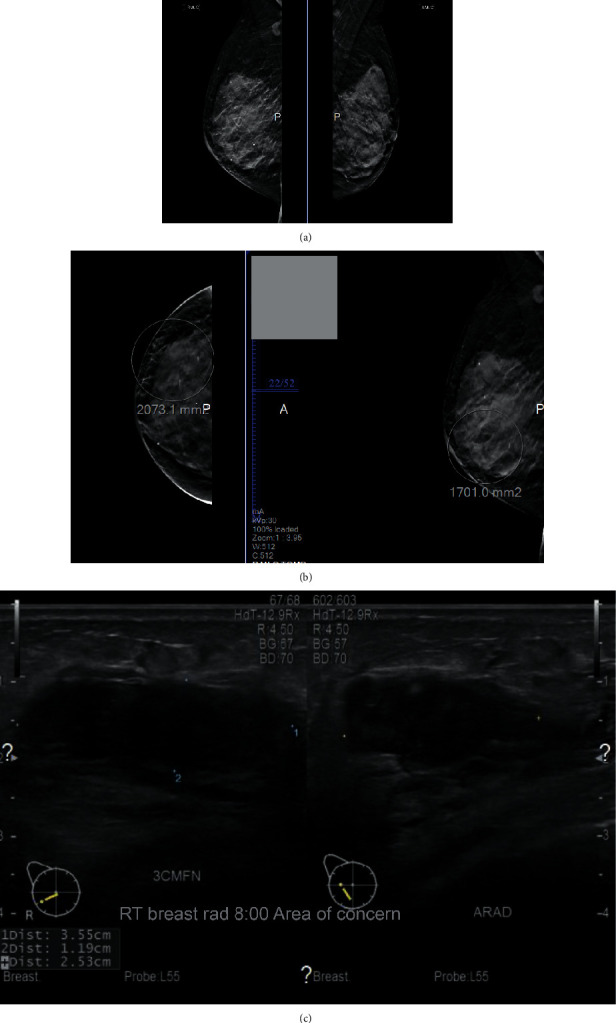
(a–c) Ill-defined mass at 8 o'clock position 3 cm from the nipple.

**Figure 2:**
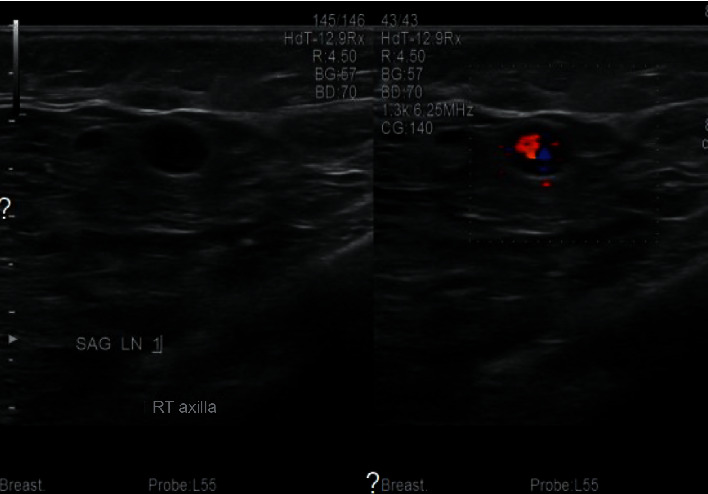
Suspicious axillary lymphadenopathy with aberrant blood flow.

**Figure 3:**
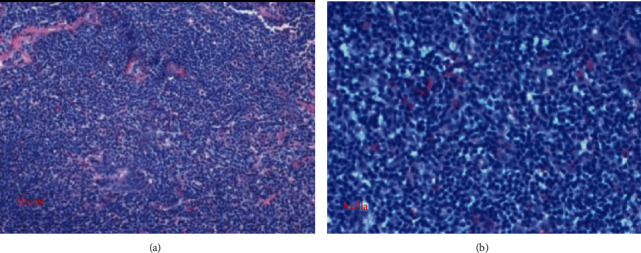
(a) Breast and (b) axillary histology.

She underwent a mammogram with 3D tomosynthesis, which revealed a 3.6 × 2.5 × 1.2 cm solid ill-defined multilobulated mass at 8 o'clock position 3 cm from the nipple (Figures 1(a)–1(c)). Additionally, two suspicious nodes in the axilla measuring 0.7 × 0.6 × 0.6 cm and 0.8 × 0.5 × 0.4 cm were identified (Figure 2). A breast ultrasound and subsequent sonographically guided core biopsy of the breast and axilla was then performed. An atypical lymphocytic infiltrate was identified in both the breast and axilla. Molecular genetic testing was performed consistent with a T-cell lymphoproliferative disorder involving both the breast and axilla (Figures 3(a) and 3(b)). There was no evidence of infiltrating ductal or lobular carcinoma. In view of this, no further breast tissue excision was performed and the patient was referred to medical oncology for further management of these lesions.

## 3. Discussion

Although PBL is an uncommon differential for a breast lump, this can be the presenting symptom as in our case and can mimic an invasive carcinoma. Clinically, it typically presents as one or multiple painless masses quite similar to breast carcinoma sometimes with skin edema and local pain [11]; ipsilateral axillary lymphadenopathy can be present in 13–50% of PBL cases [9]. Symptoms may be nonspecific and may mimic those of breast carcinoma with pain being reported to occur in 4–25% of patients [11]. Other local signs, such as nipple retraction or discharge and skin changes, are rare [11]. Similarly systemic symptoms such as weight loss, fever, and sweating are rare occurring in approximately 8–9% of cases [18]. There is a strange predominant involvement of the right breast in several series [3, 11, 19] with a right to left ratio of 2 : 1 [20]. This was also the case in our patient.

Where imaging is concerned, there are no pathognomonic mammographic features for breast lymphoma, and sometimes these lesions are only detected by ultrasound [11, 19]. While our patient's mammogram revealed an ill-defined multilobulated mass, mammography generally shows oval and high-density solitary or multiple masses with a well-circumscribed margin without calcifications or speculations [19, 21]. Irregular or partially defined margins on mammography have been reported in addition to reports of minimal and moderate spiculation as well; hence, mammographic appearances may vary [11, 20]. Diffuse increased opacity with or without skin thickening is also reported as mammographic findings in 9-33% of cases [11]. Many patients may not have any abnormalities on mammography, and ultrasound may be the only modality which detects these lesions. Hypoechoic round or oval masses are typically seen on ultrasonography sometimes even mistaken for simple cysts [21, 22]. Deviation from this pattern can exist; Lyou et al. reported a hyperechoic mass, whereas Liberman et al. in their series reported a posterior acoustical enhancement in 71% of cases. Ultrasound findings in our case were consistent with a hypoechoic lesion.

Treatment of primary breast lymphoma is largely multimodal, and a standard consensus for treatment is lacking [23]. Surgery if any is centered around diagnostic sampling rather than curative intent although literature reports a range of surgical options from biopsy only to radical mastectomy with chemotherapy and radiotherapy used as adjuvant or primary treatment.

In a large series of 465 PBL cases, Jennings et al. concluded that surgery (mastectomy) did not offer any therapeutic advantage, in fact, radiotherapy for stage 1 node-negative disease showed increased overall survival and disease-free survival (*P* = 0.002). This series also showed that axillary clearance has no role in the treatment of PBL. Also, a therapeutic regimen involving chemotherapy for stage 2 disease with nodal involvement showed improved overall and disease-free survival benefit. This series also found a positive corelation between histological grade and survival [24]. In another series of 17 cases of PBL treated at Cleveland Clinic between 1980 and 1996, improved survival was reported with multimodal therapy including surgery, radiotherapy, and chemotherapy at a median follow-up of 34 months. All these were stage 1E disease (confined to the breast or breast and ipsilateral axillary nodes [25]. Miller et al. reported significantly better disease-free and overall survival in patients treated with three cycles of chemotherapy (CHOP; Cyclophosphamide, Doxorubicin, Vincristine, Prednisolone) with field radiotherapy than those treated with CHOP alone (even at 8 cycles) for treating localized intermediate and high-grade PBL. The addition of Rituximab with CHOP increased survival significantly compared to CHOP alone [26]. The primary sites of recurrence are the axilla, contralateral breast, and CNS. The role of CNS prophylactic treatment has been extensively discussed but no consensus exists, some advocate for it, and others report that it is not justified given the rarity of the complication [27, 28]. Our patient was deemed stage 2 and underwent an ultrasound-guided biopsy as part of her diagnostic workup but we did not pursue any further surgical options. She was referred to oncology for consideration for chemotherapy after a multidisciplinary team discussion. Her metastatic workup revealed hypermetabolic activity in the known right breast lesion with a 3.4 × 1.5 cm hypermetabolic mass at the left obturator without hilar or mediastinal disease. Left femoral head avascular necrosis was noted as well as flow cytometric evidence of bone marrow involvement (5%). She is currently receiving combination immunotherapy and chemotherapy (Cytoxan, Adriamycin, and Brentuximab). Additionally, in view of the late age of presentation in our patient and the rarity of CNS recurrence, this option was discussed and the decision was to not proceed with investigations.

## 4. Conclusion

Primary breast lymphoma and specifically T-cell lymphoma is a rare differential for a breast lump, which cannot easily be differentiated from invasive ductal carcinoma. Histopathologic diagnosis is mandatory and can guide treatment. Surgical role is limited to obtaining tissue for pathological diagnosis, and primary management options include chemoradiation.

## References

[1] Takemura A., Mizukami Y., Takayama T., Taniya T., Okumura H. (2009). Primary malignant lymphoma of the breast. *Japanese Journal of Radiology*.

[2] Jinming X., Qi Z., Xiaoming Z., Jianming T. (2012). Primary non-Hodgkin's lymphoma of the breast: mammography, ultrasound, MRI and pathologic findings. *Future Oncology*.

[3] Weisman C., Liao K. T. (1972). Primary lymphoma of the breast. *Cancer*.

[4] Venizelos I. D., Tatsiou Z., Vakalopoulou S., Mandala E., Garipidou V. (2009). Primary non-Hodgkin’s lymphoma arising in an intramammary lymph node. *Leukemia & Lymphoma*.

[5] Mohamed K. E. H., Ali R. A. E. (2017). Primary breast lymphoma: a case report and review of the literature. *Clinics and Practice*.

[6] Yoshida S., Nakamura N., Sasaki Y. (2005). Primary breast diffuse large B-cell lymphoma shows a non-germinal center B-cell phenotype. *Modern Pathology*.

[7] Savage K. J., Chhanabhai M., Gascoyne R. D., Connors J. M. (2004). Characterization of peripheral T-cell lymphomas in a single North American institution by the WHO classification. *Annals of Oncology*.

[8] Jaffe E. S., Harris N. L., Stein H., Isaacson P. G. (2008). Classification of lymphoid neoplasms: the microscope as a tool for disease discovery. *Blood*.

[9] Talwalkar S. S., Miranda R. N., Valbuena J. R., Routbort M. J., Martin A. W., Medeiros L. J. (2008). Lymphomas involving the breast. A study of 106 cases comparing localized and disseminated neoplasms. *The American Journal of Surgical Pathology*.

[10] Gualco G., Bacchi C. E. (2008). B-cell and T-cell lymphomas of the breast: clinical-pathological features of 53 cases. *International Journal of Surgical Pathology*.

[11] Sabate J. M., Gomez A., Torrubia S. (2002). Lymphoma of the breast: clinical and radiologic features with pathologic correlation in 28 patients. *The Breast Journal*.

[12] Gaudet G., Friedberg J. W., Weng A., Pinkus G. S., Freedman A. S. (2009). Breast lymphoma associated with breast implants: two case-reports and a review of the literature. *Leukemia & Lymphoma*.

[13] Sahoo S., Rosen P. P., Feddersen R. M., Viswanatha D. S., Clark D. A., Chadburn A. (2003). Anaplastic large cell lymphoma arising in a silicone breast implant capsule: a case report and review of the literature. *Archives of Pathology & Laboratory Medicine*.

[14] Newman M. K., Zemmel N. J., Bandak A. Z., Kaplan B. J. (2008). Primary breast lymphoma in a patient with silicone breast implants: a case report and review of the literature. *Journal of Plastic, Reconstructive & Aesthetic Surgery*.

[15] Fritzsche F. R., Pahl S., Petersen I. (2006). Anaplastic large-cell non-Hodgkin’s lymphoma of the breast in periprosthetic localisation 32 years after treatment for primary breast cancer—a case report. *Virchows Archiv*.

[16] Kraemer D. M., Tony H. P., Gattenlöhner S., Müller J. G. (2004). Lymphoplasmacytic lymphoma in a patient with leaking silicone implant. *Haematologica*.

[17] Roden A. C., Macon W. R., Keeney G. L., Myers J. L., Feldman A. L., Dogan A. (2008). Seroma-associated primary anaplastic large-cell lymphoma adjacent to breast implants: an indolent T-cell lymphoproliferative disorder. *Modern Pathology*.

[18] Kuper-Hommel M. J. J., Snijder S., Janssen-Heijnen M. L. G. (2003). Treatment and survival of 38 female breast lymphomas: a population-based study with clinical and pathological reviews. *Annals of Hematology*.

[19] Lyou C. Y., Yang S. K., Choe D. H., Lee B. H., Kim K. H. (2007). Mammographic and sonographic findings of primary breast lymphoma. *Clinical Imaging*.

[20] Surov A., Holzhausen H. J., Wienke A. (2012). Primary and secondary breast lymphoma: prevalence, clinical signs and radiological features. *The British Journal of Radiology*.

[21] Yang W. T., Lane D. L., Le-Petross H. T., Abruzzo L. V., Macapinlac H. A. (2007). Breast lymphoma: imaging findings of 32 tumors in 27 patients. *Radiology*.

[22] Liberman L., Giess C. S., Dershaw D. D., Louie D. C., Deutch B. M. (1994). Non-Hodgkin lymphoma of the breast; imaging characteristics and correlation with histopathologic findings. *Radiology*.

[23] Ganjo K., Advani R., Marippan M. R. (2007). Breast non Hodgkins lymphoma. *Cancer*.

[24] Jennings W. C., Baker R. S., Murray S. S. (2007). Primary breast lymphoma. *Annals of Surgery*.

[25] Lyons J. A., Myles J., Pohlman B., Macklis R. M., Crowe J., Crownover R. L. (2000). Treatment of prognosis of primary breast lymphoma: a review of 13 cases. *American Journal of Clinical Oncology*.

[26] Miller M., Danlberg S., Cassady J. R. (2006). PBL, long term outcome and prognosis. *Leukemia & Lymphoma*.

[27] Kusano Y., Nishimura N., Ueda K., Yokoyama M., Terui Y., Hatake K. (2013). Intrathecal prophylaxis might be required for primary and secondary breast lymphoma to prevent central nervous system relapse. *Blood*.

[28] Cheah C. Y., Campbell B. A., Seymour J. F. (2014). Primary breast lymphoma. *Cancer Treatment Reviews*.

